# Predictive factors of treatment outcomes after percutaneous ablation of hepatocellular carcinoma in the caudate lobe: a retrospective study

**DOI:** 10.1186/s12885-019-5881-0

**Published:** 2019-07-16

**Authors:** Baoxian Liu, Jianting Long, Wei Wang, Tongyi Huang, Xiaohua Xie, Shuling Chen, Guangliang Huang, Chunlin Jiang, Jieyi Ye, Haiyi Long, Xiaoyan Xie, Ming Kuang

**Affiliations:** 1 Division of Interventional Ultrasound, Department of Medical Ultrasound, The First Affiliated Hospital, Sun Yat-sen University, Guangzhou, 510080 China; 2 Department of Oncology, The First Affiliated Hospital, Sun Yat-sen University, Guangzhou, 510080 China; 3 Department of Liver Surgery, The First Affiliated Hospital, Sun Yat-sen University, Guangzhou, 510080 China

**Keywords:** Percutaneous ablation therapy, Hepatocellular carcinoma, Caudate lobe, Treatment outcomes

## Abstract

**Background:**

Hepatocellular carcinomas (HCC) arising in the caudate lobe is rare and the treatment is difficult. The aim of this study is to summarize the experience of ultrasound-guided percutaneous ablation therapy for HCC located in the caudate lobe and to investigate the predictive factors of the treatment outcomes.

**Methods:**

From August 2006 to June 2017, 73 patients (63 males and 10 females; mean age, 54.9 ± 11.6 years; age range, 25–79 years) with 73 caudate lobe HCCs (mean size, 2.6 ± 1.1 cm; size range, 1.0–5.0 cm) were treated with percutaneous ablation, including 33 patients with radiofrequency ablation (RFA), 23 patients with ethanol ablation (EA), and 17 patients with combination of RFA and EA. The treatment outcome and survival after ablation for caudate lobe HCC were assessed and the predictive factors were calculated by univariate and multivariate analyses.

**Results:**

A total of 72 patients achieved complete ablation after the first or second session of ablation. The treatment effectiveness was 98.6% (72/73). During the follow-up, 16 tumors developed local tumor progression (LTP) and a total of 61 patients (61/73, 83.6%) were detected distant recurrence (DR). According to univariate and multivariate analyses, tumor size > 2 cm (hazard ratio[HR] = 3.667; 95% confidence interval[CI], 1.043–12.889; *P* = 0.043) was a significant prognostic factor of LTP after ablation for HCC in the caudate lobe, while tumor number (HR = 2.245; 95%CI, 1.168–4.317; *P* = 0.015) was a significant prognostic factor of DR. The mean overall survival time after ablation was 28.7 ± 2.8 months, without independent predictive factors detected. Four patients (4/73, 5.5%) were detected treatment-related major complications, without independent predictive factor detected.

**Conclusion:**

Ultrasound-guided percutaneous ablation is a feasible treatment for a selected case with HCC in the caudate lobe. Tumor size > 2 cm increases the risk of LTP and intrahepatic tumor number is associated with DR after ablation.

## Background

Hepatocellular carcinoma (HCC) in the caudate lobe is rare. The treatment of it is difficult, because the caudate lobe is located deeply between the hepatic hilum and the inferior vena cava. Surgical resection his considered the curative treatment for caudate lobe HCC, but resection of the caudate lobe is associated with considerable technical difficulty and is challenging for the hepatic surgeon [1, 2].

Percutaneous ablation as a minimally invasive technique is recommended for small HCCs in patients with preserved liver function reserve, according to the guidelines established by American Association for the Study of Liver Disease (AASLD) and European Association for the Study of Liver (EASL) [3]. Among the various local ablation techniques, radiofrequency ablation (RFA) is the most commonly used modality [4]. It has been reported that RFA can obtain a complete response comparable to the liver resection [5, 6]. Ethanol ablation (EA) is also an effective treatment that has been widely used in patients with small tumors, especially for those at high-risk locations [7]. Moreover, the combination of RFA and EA (RFA-EA) can overcome the limitations of RFA alone [8] and EA alone [9], resulting in improved overall survival (OS) and reduced the risk of local tumor progression (LTP) without increasing major complications [10].

Despite the increased application of percutaneous ablation for HCC, caudate lobe ablation has only been initially described. The deep location and specific anatomic features of the caudate lobe render ablation technically challenging [11–14]. In addition, the complex arterial blood supply of the caudate lobe further complicates the ablation therapy [15, 16]. Although some studies have reported that ablation is an effective treatment for HCC in the caudate lobe, the vast majority of data are reported from small series. As it reported, the LTP rate of caudate lobe ablation is higher than the non-caudate lobe [13]. The predictive factors of local treatment outcomes for caudate lobe tumor are important but still unclear.

The aim of our study is to summarize our experience of ultrasound-guided percutaneous ablation therapy for HCC located in the caudate lobe and investigate the predictive factors of treatment outcomes.

## Methods

This was a retrospective study and performed according to the guidelines of the Helsinki Declaration. The study was approved by the Ethical Committee of the First Affiliated Hospital of Sun Yat-sen University and written informed consent was obtained from all patients.

### Patients

**Fig. 1:**
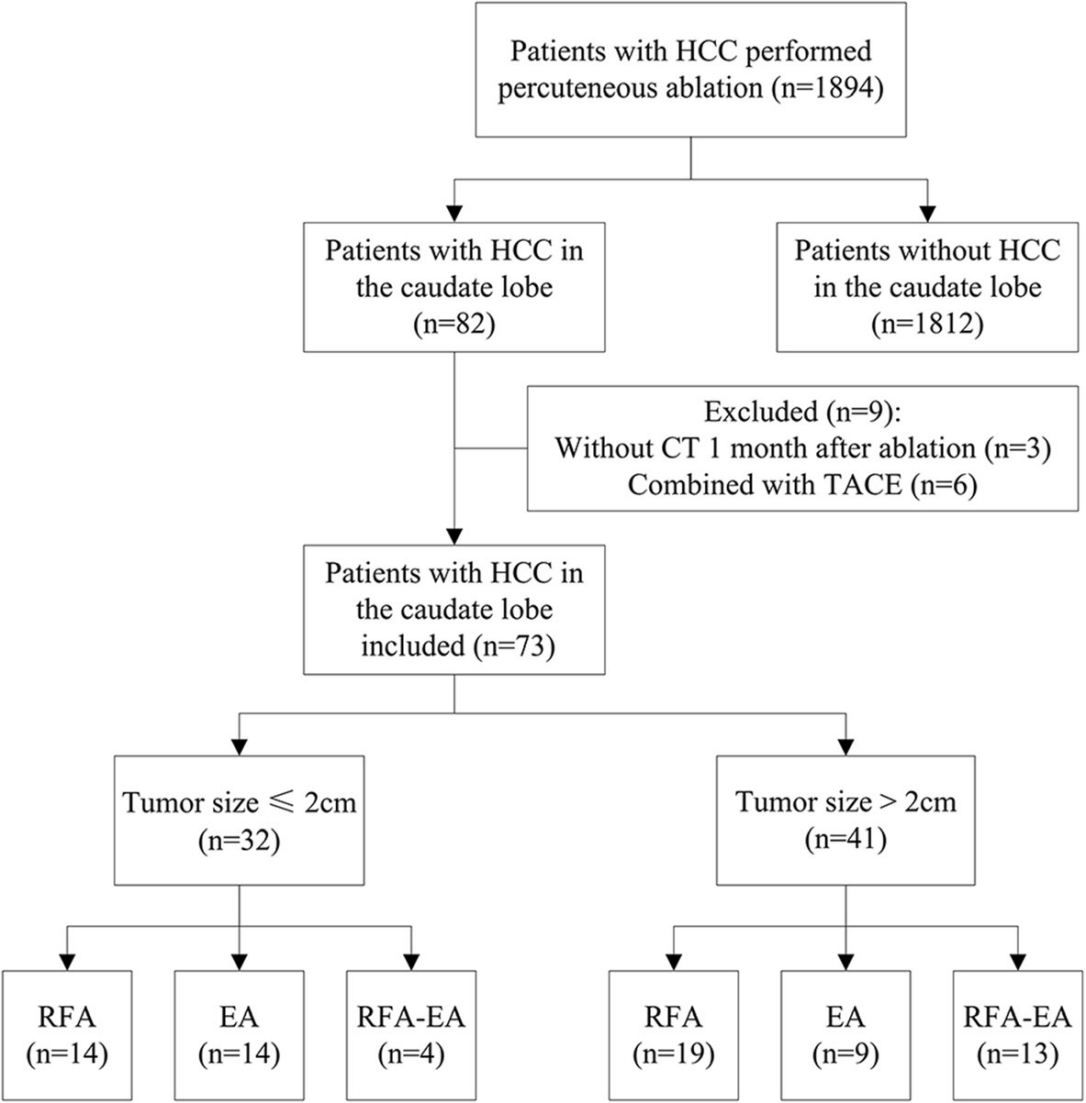
Flow diagram of the study

From August 2006 to June 2017, 1894 patients with HCCs underwent ultrasound-guided percutaneous ablation therapy. Among them, 82 patients had tumors in the caudate lobe and the remaining 1812 patients had no tumor in the caudate lobe. Nine patients with caudate lobe tumors were excluded due to the lack of complete data of treatment in 3 and combined with transcatheter arterial chemoembolization (TACE) in 6. Finally, a total of 73 patients (63 males and 10 females; mean age, 54.9 ± 11.6 years; age range, 25–79 years) with 73 HCCs in the caudate lobe were included (Fig. 1). The mean tumor size was 2.6 ± 1.1 cm (range: 1.0–5.0 cm). HCC was diagnosed by ultrasound-guided biopsy by using an 18 gauge needle (Bard Corporation, State of New Jersey, United States) or by the diagnostic criteria published by the EASL (i.e., typical features of HCC or positive findings on one imaging study together with an alpha-fetoprotein (AFP) level of > 400 ng/mL) [17].

#### The inclusion criteria were as follows

- Adult (18–80 years) with HCC in the caudate lobe and refused surgical recession;
- Tumor number not more than five and the largest tumor size not larger than 5.0 cm;
- Liver function status at Child-Pugh class A or B;
- East Coast Oncology Group (ECOG) performance status value 0 or 1;
- No severe coagulopathy (e.g. platelets ≥50,000/ml, prothrombine time ratio ≥ 50%).
- Available medical records and/or imaging studies.

#### Exclusion criteria of ablation included

- Presence of vascular invasion and extrahepatic metastases at preprocedure imaging study;
- Ongoing anticoagulant treatment that cannot be stopped;
- Previous treatment such as TACE for caudate lobe tumor.

### Treatment protocols

A panel discussion with our multidisciplinary treatment team including surgeons, radiologists, oncologists and pathologists was performed to make a decision regarding the optimal treatment modality. RFA was recommended as the first choice for the tumors with sufficient safety margin for thermal ablation. For tumors ≤2 cm without sufficient safety margin, EA alone was chosen. For tumors without sufficient safety margin and larger than 2 cm, combination of RFA and EA was recommended. Sufficient safety margin was defined as tumor located > 5 mm from important structures, such as inferior vena cava, hepatic vein, and liver capsule determined by pre-treatment computed tomography (CT) images.

### Percutaneous ablation therapy

All procedures were performed by two experienced authors (X.Y.X. and M.K., both had experience in tumor ablation more than 10 years) with the real-time ultrasound guidance using Aloka α10 ultrasound scanner (Aloka Inc., Tokyo, Japan) with a 2.0–6.0 MHz puncture probe, Acuson Sequoia 512 ultrasound scanner (Siemens Medical Solutions, Mountain View, CA) with a 1.0–4.0 MHz puncture probe, Aplio 500 ultrasound scanner (Toshiba Medical Systems, Tokyo, Japan) with a 1.9–6.0 MHz puncture probe, or Aixplorer ultrasound scanner (SuperSonic Imagine, Aix en Provence, France) with a 1.0–6.0 MHz probe.

Three approaches were used to insert the needle into the caudate lobe: the left lobe approach (LA), the right intercostal approach (RA) through the right hepatic lobe, and combination approach of LA and RA. Generally, LA was used for HCCs in the Spiegel lobe and RA was used for HCCs in the paracaval portion and in the caudate process. When a vessel that could potentially be injured by the needle was present in the extrahepatic space between the lateral segment of the liver and the caudate lobe, RA was chosen even the tumor was located in the Spiegel lobe.

#### EA

After administration of local anesthesia (5 ml of 1% lidocaine), a 21-gauge needle (Hakko Co., Ltd., Nagano, Japan) was inserted into the low-center of the tumor under US guidance, and the tip of the needle was positioned at the inferior aspect of the tumor. Ethanol was injected two to four sessions separate days, until the entire tumor appeared completely hyperechoic. The general guideline for the necessary amount of injected ethanol was calculated according to the numerical expression V = (4/3) [π (D/2 + 0.5) ^3^], in which V (in mL) is the volume of ethanol and D (in cm) is the diameter of the tumor. After the completion of ethanol injection, the needle was left in place for 1–2 min before it was withdrawn.

#### RFA

RFA was performed with conscious analgesic sedation (intravenous administration of 0.1 mg of fentanyl, 5 mg of droperidol and 0.1 mg of tramadol hydrochloride) and local anaesthesia (5 ml of 1% lidocaine). Vital signs were continuously monitored during the procedure. RFA devices used in this study were LeVeen electrodes (Boston Scientific, Natick, MA), Starburst XL electrodes (RITA Medical Systems, Mountain View, CA) and Cool-tip electrodes (Valleylab, Boulder, CO). The selection of device was based on the size and location of the tumor. The number of electrodes to be used in ablation was determined based on the tumor size, shape, and location with the aim of achieving an ablative margin at least 0.5 cm beyond the tumor boundary. If necessary, after the first application, the needle was pulled out 1 cm and a second application was started. After an ablation was completed, the needle track was carefully treated with the electrode by retracting by 1 cm increments to prevent bleeding and tumor seeding.

#### RFA-EA

For patients scheduled to perform RFA-EA, the RFA needle was firstly inserted into the target tumor. Afterwards, a 21-gauge needle was placed immediately adjacent to the radiofrequency needle from another access path for ethanol injection, with the needle tip positioned at the bottom of the tumor. RFA started 3–5 min after the completion of EA. The RFA procedure was performed as described above.

After the ablation, patients were hospitalized for 1–2 days, unless there were complications. Complications were defined and assessed according to SIR classification [18]. Major complication was defined as an event that leads to substantial morbidity and disability that increases the level of care, or results inhospital admission or substantially lengthens the hospital stay. All other complications were considered minor.

### Treatment outcomes and follow-up

One month after ablation, contrast-enhanced CT was performed to evaluate the technical effectiveness. Complete ablation (CA) was defined as a complete non-enhancement of treated tumor on contrast-enhanced CT. In the case with viable residual tumor, incomplete ablation (ICA) was defined and additional session of ablation was given. If the residual tumor was still viable after the additional session, percutaneous ablation therapy was considered a failure and the patients were referred to other therapies. The ablative margin (AM) between the index tumor and the ablated zone was confirmed by contrast-enhanced imaging obtained before and after ablation in a side-by-side manner by two authors (B.X.L. and W.W, with an experience of tumor ablation of 6 years and 12 years, respectively) with chart.

Thereafter, all patients were followed up by conventional ultrasound / contrast-enhanced ultrasound (CEUS), serum AFP and liver function 3 monthly for the first 2 years, then 6 monthly from 2 to 5 years and 12 monthly after 5 years. Contrast-enhanced CT or Magnetic Resonance Imaging (MRI) scanning was performed if suspicious recurrence was detected on CEUS. We evaluated potential outcome predictors with the three primary endpoints: treatment-related major complications, ICA and LTP. Secondary endpoints assessed by distant recurrence (DR) and OS. LTP was defined as the reappearance of enhancing tumor tissue adjacent to the ablated zone after achievement of ablation success [19].

### Statistical analysis

Continuous variables were expressed as mean ± standard deviation and categorical variables were expressed as rate with 95% confidence intervals [CI].

For suspicious risk factors associated with ICA and treatment-related major complications such as gender, age, histological pattern, hepatitis, liver cirrhosis, ECOG performance status, Child-Pugh grade, platelets [PLT], prothrombin time [PT], albumin [ALB], alanine aminotransferase [ALT], total bilirubin [TB], tumor number, surgery history of liver, location, tumor size, puncture approach, treatment strategy, etc., univariate associations between individual variables were tested by chi-square test or Fisher’s exact test as appropriate. Logistic regression analysis was used for multivariate analysis. Variables with *P* values of < 0.10 in the univariate analysis were chosen as variables for multivariate analysis.

For suspicious risk factors of LTP such as gender, age, histological pattern, hepatitis, liver cirrhosis, ECOG performance status, Child-Pugh grade, PLT, PT, ALB, ALT, TB, tumor number, surgery history of liver, location, tumor size, puncture approach, treatment strategy and AM, the Kaplan-Meier method was used for univariate analysis whereas the Cox proportional hazards regression model was used for multivariate analysis. Variables with *P* values < 0.10 in the univariate analysis were chosen as variables for multivariate analysis.

All the statistical analyses were carried out using SPSS version 16.0 (Chicago, IL, USA). A two-tailed *P* value < 0.05 was considered as statistical significant difference.

## Results

### Patients and tumor profile

**Fig. 2:**
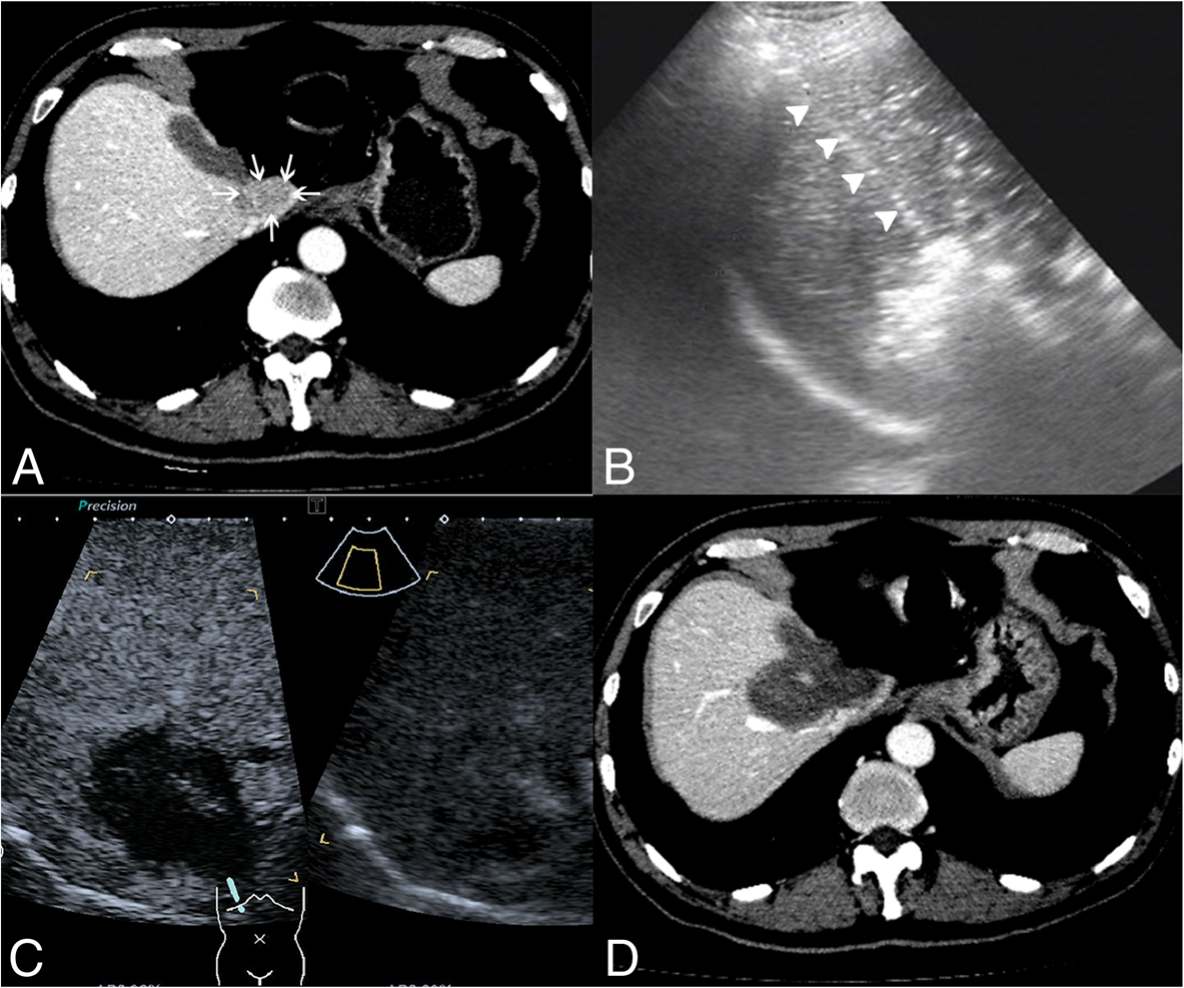
Images of a patient with recurrent hepatocellular carcinoma (HCC) in the caudate lobe treated by percutaneous radiofrequency ablation (RFA). **a** transverse contrast-enhanced computer tomography (CT) before treatment shows the tumor in the caudate lobe (arrow); **b** tumor appears completely hyperechoic after the beginning of RFA, and the electrode (arrowhead) is clearly showed on the ultrasound image; **c** CEUS image obtained 1 month after RFA shows the tumor completely ablated; **d** contrast-enhanced CT image obtained 1 month after RFA shows the tumor completely ablated

Seventy-three patients with 73 HCCs located in caudate lobe were performed percutaneous ablation during 10 years in our hospital (Fig. 2). Among them, 36 tumors were located in the paracaval portion, seven in the caudate process, and 30 in the Spiegel lobe. RFA was performed on 33 patients, EA was performed on 23 patients, and RFA-EA was performed on 17 patients. There were 32 caudate lobe tumors with size ≤2 cm and 41 caudate lobe tumors with size > 2 cm. The mean tumor size was 2.6 ± 1.1 cm (range: 1.0–5.0 cm) (Table 1).

**Table 1:** Demographic data and tumor characteristics

Parameter	*n* = 73
Gender (M/F)	
Male	63
Female	10
Age (year)^a^	54.9 ± 11.6 (25–79)
Etiology	
HBV	55
HCV	4
Others	14
Tumor type	
Naïve	19
Recurrent	54
Surgery history of liver	
Yes	34
No	37
Liver cirrhosis	
Yes	51
No	22
Antiviral treatment	
Yes	42
No	31
ECOG performance status (0/1)	
0	71
1	2
Child-Pugh	
A	67
B	6
Tumor stage of primary HCC	
BCLC A	61
BCLC B	12
Laboratory data #	
AFP (μg/L)	1021.4 ± 2216.2 (1.85–8034.17)
PLT (× 10^9^/L)	174.1 ± 71.4 (69–345)
PT (s)	12.6 ± 1.1 (10.5–17.1)
ALB (g/L)	40.4 ± 4.8 (27–49.7)
ALT (IU/L)	29.9 ± 16.9 (7–84)
TB (mol/L)	13.6 ± 5.7 (5.4–30.8)
Tumor number	
Single	38
Multiple	33
Location	
Paracaval portion	36
Caudate process	7
Spiegel’s lobe	30
Tumor size (cm)	
≤ 2 cm	32
> 2 cm	41
Treatment strategy	
RFA	33
EA	23
RFA-EA	17
Ablative margin	
< 5 mm	66
≥ 5 mm	7

*HBV* hepatitis B virus, *HCV* hepatitis C virus, *ECOG* East Coast Oncology Group, *BCLC* Barcelona Clinic Liver Cancer, *AFP* alpha-fetoprotein, *PLT* platelets, *PT* prothrombin time, *ALB* albumin, *ALT* alanine aminotransferase, *TB* total bilirubin, *EA* ethanol ablation, *RFA* radiofrequency ablation, *RFA-EA* combination of RFA and EA

^a^Data are means ± standard deviations (range)

For HCCs treated by EA, the mean volume of injected ethanol was 18.0 ± 9.0 ml (range, 7–40 ml). For HCCs treated by RFA-EA, the mean ethanol volume was 10.9 ± 4.7 ml (range, 2–30 ml), which was significantly less than that in the EA group (*P* = 0.011).

### Treatment response

A total of eight HCCs in the caudate lobe were detected residual tumors. Sixty-five out of 73 HCCs in the caudate achieved CA after the first session. By univariate analysis, only treatment strategy of EA versus RFA or RFA-EA was associated ICA after the first session of ablation for caudate lobe tumor (*P* = 0.029). Variables of histological pattern (*P* = 0.088), treatment strategy of RFA versus EA versus RFA-EA (*P* = 0.055), and treatment strategy of RFA with or without EA versus EA (*P* = 0.029) entered multivariate analysis. Multivariate Cox proportional hazards regression analysis showed that treatment strategy of EA alone (hazard ratio [HR] = 5.031; 95%CI, 1.299–19.481; *P* = 0.019) was significantly independent prognostic factors of ICA in the patients with HCC in the caudate lobe (Table 2).

**Table 2:** Univariate and multivariate analysis of predictors of incomplete ablation after the first session of caudate lobe ablation

Factors		Univariate	Multivariate		
		*P* value	HR	95%CI	*P* value
Gender(M/F)		0.686			
Age(≤60/>60y)		0.716			
Tumor type (naive/recurrent)		0.088	–	–	–
Etiology (Hepatitis/others)		0.508			
Type (Naïve/recurrent)		0.563			
Surgery history of liver (Y/N)		0.566			
Liver cirrhosis (Y/N)		0.382			
Antiviral treatment (Y/N)		0.864			
ECOG performance status (0/1)		0.811			
Child-Pugh (A/B)		0.221			
BCLC stage of primary HCC (A/B)		0.231			
AFP (≥400/< 400 μg/L)		0.197			
PLT (≥100/< 100 × 10^9^/L)		0.374			
PT (≤14/> 14 s)		0.221			
ALB (≤35/> 35 g/L)		0.354			
ALT (≤40/> 40 IU/L)		1.000			
TB (≤17.1/> 17.1 mol/L)		0.401			
Tumor number (single /multiple)		0.519			
Location (Paracaval portion/ Caudate process/ Spiegel’s lobe)		0.807			
Tumor size (≤2 cm/> 2 cm)		0.907			
Puncture approach (LA/ RA/ combination approach)		0.177			
Treatment strategy	RFA/ EA/ RFA-EA	0.055	–	–	–
	EA/RFA or RFA-EA	0.029	5.031	1.299–19.481	0.019
	RFA or EA/RFA-EA	0.721			

*HBV* hepatitis B virus, *HCV* hepatitis C virus, *ECOG* East Coast Oncology Group, *BCLC* Barcelona Clinic Liver Cancer, *AFP* alpha-fetoprotein, *PLT* platelets, *PT* prothrombin time, *ALB* albumin, *ALT* alanine aminotransferase, *TB* total bilirubin, *LA* left lobe approach, *RA* right intercostal approach, *EA* ethanol ablation, *RFA* radiofrequency ablation, *RFA-EA* combination of RFA and EA

Seven of them (7/8, 87.5%) received additional session of ablation and achieved CA. Therefore, a total of 72 (72/73, 98.6%) tumors in the caudate lobe from 72 patients were successfully ablated. Therefore, the treatment effectiveness was 98.6% (72/73). The remaining one patient with HCC only received sorafenib treatment for the residual tumor due to the portal vein tumor thrombi and intrahepatic distance recurrence plus extrahepatic metastasis. This patient received EA for the caudate lobe HCC, which was 3.9 cm in the maximum diameter. Among patients with CA for caudate lobe tumor, 7 tumors (7/72, 9.7%) achieved AM ≥5 mm and 65 tumors (65/72, 90.3%) showed AM < 5 mm.

### LTP

**Fig. 3:**
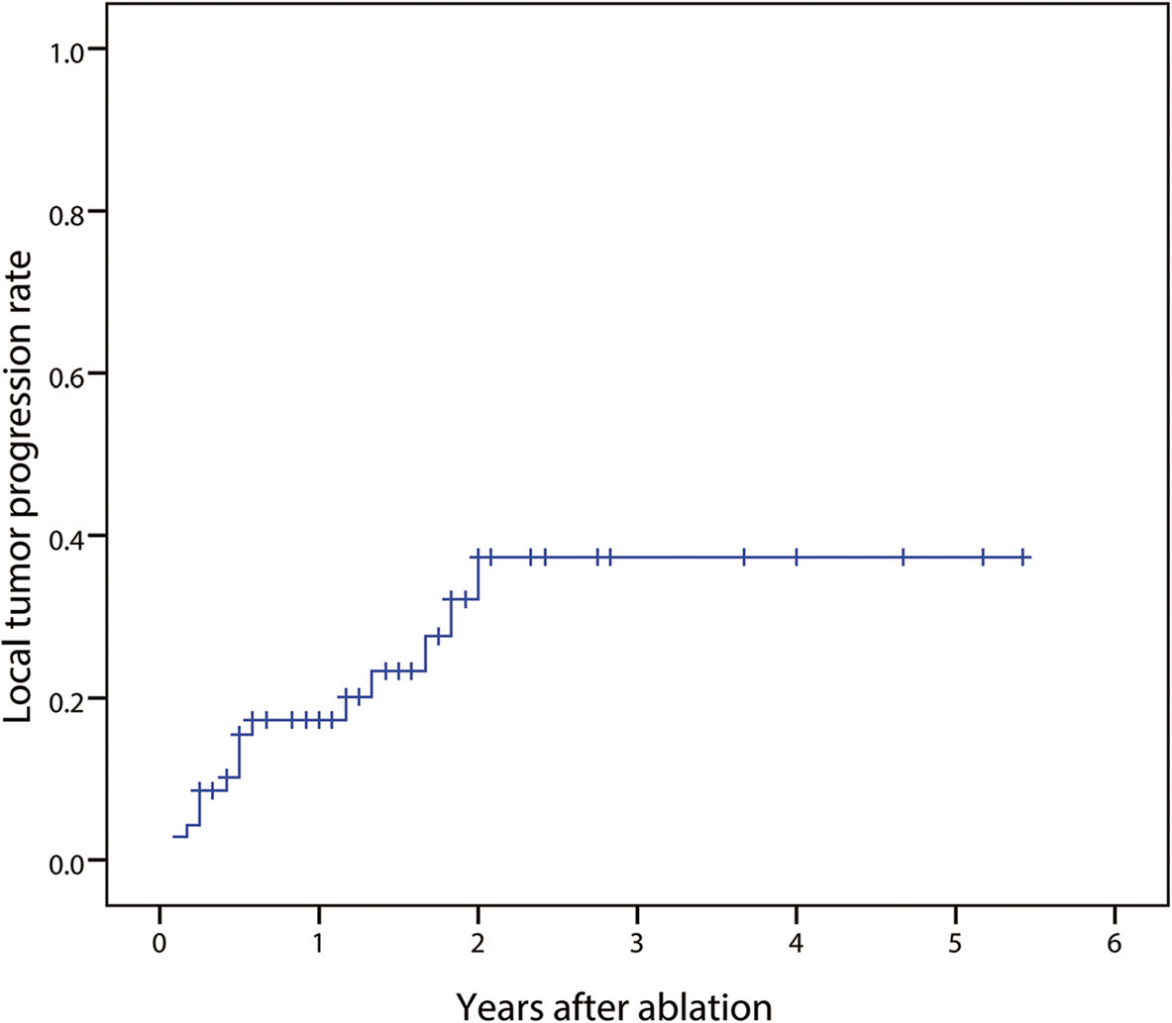
Local tumor progression of caudate lobe tumor after ablation

All the patients were entered follow-up and the patients with CA were observed for LTP (*n* = 72). The mean observation period was 18 months (range, 3–65 months). During the follow-up period, a total of 16 tumors in the caudate lobe (16/72, 22.2, 95% CI: 13.8, 32.9%) developed LTP after 2 to 24 months (mean, 8.8 months; median, 10.5 months). The 1-, 3-, and 5-year LTP rates were 17.3, 37.3, and 37.3%, respectively (Fig. 3). There was no significant difference in the LTP between patients achieving CA after the first session and patients defined as ICA and underwent additional session (*P* = 0.427). In regard to LTP, eight patients received RFA, four patients received EA, and one patient received hepatic resection. The remaining three patients were treated by TACE due to intrahepatic multiple recurrences.

**Fig. 4:**
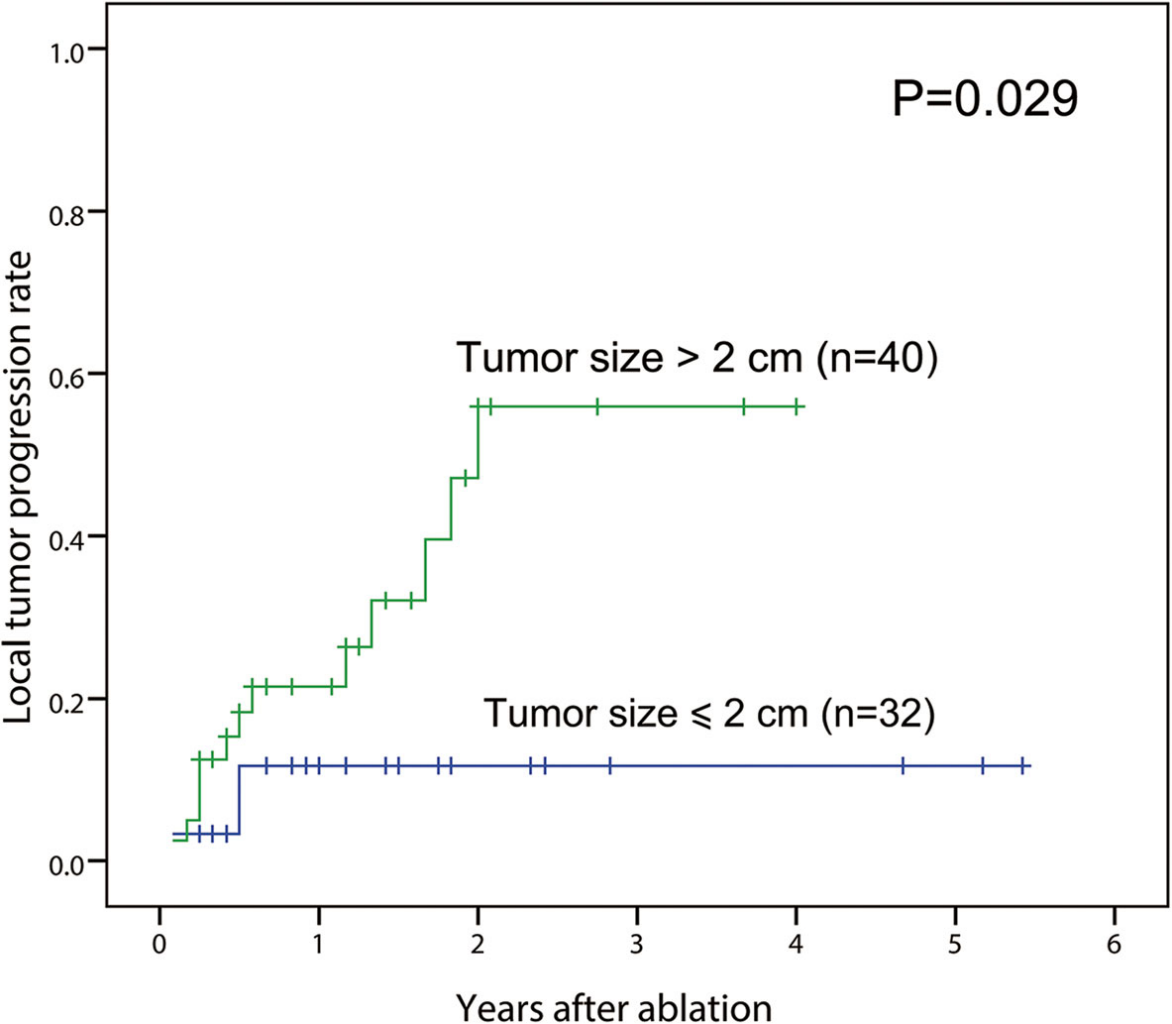
Comparison of local tumor progression of caudate lobe tumor after ablation according to tumor size

Based on tumor size, the 1- and 3-year LTP rates in the tumor size ≤2 cm group were 11.5 and 11.5%, respectively. Meanwhile, the 1- and 3-year LTP rates in the tumor size > 2 cm group were 21.5, and 56.0%, respectively (*P* = 0.029). On the other hand, 24.6% (16/65) patients with AM < 5 mm were detected LTP, whereas none patient (0/7) with AM ≥5 mm had LTP, without significant difference (*P* = 0.336). According to univariate analysis, only tumor size (≤ 2 cm versus > 2 cm) was associated LTP (*P* = 0.029) (Fig. 4). Variables of tumor size (*P* = 0.029), treatment strategy of RFA versus EA versus RFA-EA (*P* = 0.099), and treatment strategy of RFA or EA versus RFA-EA (*P* = 0.054) entered the multivariate analysis. Multivariate Cox proportional hazards regression analysis showed that tumor size > 2 cm (HR = 3.667; 95% CI, 1.043–12.889; *P* = 0.043) was significant prognostic factor of LTP after ablation of HCC in the caudate lobe (Table 3).

**Table 3:** Univariate and multivariate analysis of predictors of LTP after caudate lobe ablation

Factors		Univariate	Multivariate		
		*P* value	HR	95%CI	*P* value
Gender(M/F)		0.401			
Age(≤60/>60y)		0.811			
Tumor type (naive/recurrent)		0.846			
Surgery history of liver (Y/N)		0.655			
Etiology (Hepatitis/others)		0.821			
Liver cirrhosis (Y/N)		0.499			
Antiviral treatment (Y/N)		0.539			
ECOG performance status (0/1)		0.768			
Child-Pugh (A/B)		0.998			
BCLC stage of primary HCC (A/B)		0.661			
AFP (≥400/< 400 μg/L)		0.432			
PLT (≥100/< 100 × 10^9^/L)		0.530			
PT (≤14/> 14 s)		0.877			
ALB (≤35/> 35 g/L)		0.280			
ALT (≤40/> 40 IU/L)		0.162			
TB (≤17.1/> 17.1 mol/L)		0.616			
Tumor number (single /multiple)		0.336			
Location (Paracaval portion/ Caudate process/ Spiegel’s lobe)		0.466			
Tumor size (≤2 cm /> 2 cm)		0.029	3.667	1.043–12.889	0.043
Puncture approach (LA/ RA/ combination approach)		0.921			
Treatment strategy	RFA/ EA/ RFA-EA	0.099	–	–	–
	EA/RFA or RFA-EA	0.223			
	RFA or EA/RFA-EA	0.054	–	–	–
AM (≥ 5 mm / < 5 mm)		0.336			

*HBV* hepatitis B virus, *HCV* hepatitis C virus, *ECOG* East Coast Oncology Group, *BCLC* Barcelona Clinic Liver Cancer, *AFP* alpha-fetoprotein, *PLT* platelets, *PT* prothrombin time, *ALB* albumin, *ALT* alanine aminotransferase, *TB* total bilirubin, *LA* left lobe approach, *RA* right intercostal approach, *EA* ethanol ablation, *RFA* radiofrequency ablation, *RFA-EA* combination of RFA and EA, *AM* ablative margin

### DR and OS

After ablation of caudate lobe HCC, 61 patients (61/73, 83.6%) were detected DR, including intrahepatic recurrences in 26 patients, extrahepatic recurrences in seven patients, and intrahepatic plus extrahepatic recurrences in 28 patients. The 1-, 2-, and 3-year DR rates were 75.8, 89.4 and 91.8%, respectively. According to univariate analysis and multivariate analysis, only tumor number (HR = 2.245; 95%CI, 1.168–4.317; *P* = 0.015) was a significant prognostic factor of DR after ablation (Table 4).

**Table 4:** Univariate and multivariate analysis of predictors of distant recurrence after caudate lobe ablation in patients with HCC

Factors		Univariate	Multivariate		
		*P* value	HR	95%CI	*P* value
Gender(M/F)		0.946			
Age(≤60/>60y)		0.317			
Tumor type (naïve/recurrent)		0.535			
Surgery history of liver (Y/N)		0.341			
Etiology (Hepatitis/others)		0.915			
Liver cirrhosis (Y/N)		0.927			
Antiviral treatment (Y/N)		0.143			
ECOG performance status (0/1)		0.823			
Child-Pugh (A/B)		0.490			
BCLC stage of primary HCC (A/B)		0.654			
AFP (> 400/≤400 μg/L)		0.601			
PLT (≥100/< 100 × 109/L)		0.267			
PT (≤14/> 14 s)		0.486			
ALB (≤35/> 35 g/L)		0.195			
ALT (≤40/> 40 IU/L)		0.969			
TB (≤17.1/> 17.1 mol/L)		0.591			
Tumor number (single/multiple)		0.008	2.245	1.168–4.317	0.015
Location (Paracaval portion/ Caudate process/ Spiegel’s lobe)		0.954			
Tumor size (≤2 cm /> 2 cm)		0.878			
Puncture approach (LA/ RA/ combination approach)		0.571			
Treatment strategy	RFA/ EA/ RFA-EA	0.546			
	EA/RFA or RFA-EA	0.787			
	RFA or EA/RFA-EA	0.768			
AM (≥ 5 mm / < 5 mm)		0.223			

*HBV* hepatitis B virus, *HCV* hepatitis C virus, *ECOG* East Coast Oncology Group, *BCLC* Barcelona Clinic Liver Cancer, *AFP* alpha-fetoprotein, *PLT* platelets, *PT* prothrombin time, *ALB* albumin, *ALT* alanine aminotransferase, *TB* total bilirubin, *LA* left lobe approach, *RA* right intercostal approach, *EA* ethanol ablation, *RFA* radiofrequency ablation, *RFA-EA* combination of RFA and EA, *AM* ablative margin

At the end of follow-up, 47 patients died of liver failure or tumor recurrence. The mean survival time after ablation for caudate lobe HCC was 28.7 ± 2.8 months, with the 1-, 3-, and 5-year OS rates at 79.4, 43.7, and 11.2%, respectively. No factor was the independent prognostic factor of OS after ablation (Table 5).

**Table 5:** Univariate and multivariate analysis of predictors of overall survival outcome after caudate lobe ablation in patients with HCC

Factors		Univariate	Multivariate		
		*P* value	HR	95%CI	*P* value
Gender(M/F)		0.880			
Age(≤60/>60y)		0.917			
Tumor type (naïve/recurrent)		0.137			
Surgery history of liver (Y/N)		0.564			
Etiology (Hepatitis/others)		0.315			
Liver cirrhosis (Y/N)		0.229			
Antiviral treatment (Y/N)		0.122			
ECOG performance status (0/1)		0.877			
Child-Pugh (A/B)		0.292			
BCLC stage of primary HCC (A/B)		0.873			
AFP (≥400/< 400 μg/L)		0.283			
PLT (≥100/< 100 × 109/L)		0.529			
PT (≤14/> 14 s)		0.901			
ALB (≤35/> 35 g/L)		0.254			
ALT (≤40/> 40 IU/L)		0.518			
TB (≤17.1/> 17.1 mol/L)		0.886			
Tumor number (single /multiple)		0.101			
Location (Paracaval portion/ Caudate process/ Spiegel’s lobe)		0.069			
Tumor size (≤2 cm /> 2 cm)		0.720			
Puncture approach (LA/ RA/ combination approach)		0.809			
Treatment strategy	RFA/ EA/ RFA-EA	0.960			
	EA/RFA or RFA-EA	0.807			
	RFA or EA/RFA-EA	0.529			
AM (≥ 5 mm / < 5 mm)		0.189			
LTP (Y/N)		0.890			
Major complication (Y/N)		0.172			

*HBV* hepatitis B virus, *HCV* hepatitis C virus, *ECOG* East Coast Oncology Group, *BCLC* Barcelona Clinic Liver Cancer, *AFP* alpha-fetoprotein, *PLT* platelets, *PT* prothrombin time, *ALB* albumin, *ALT* alanine aminotransferase, *TB* total bilirubin, *LA* left lobe approach, *RA* right intercostal approach, *EA* ethanol ablation, *RFA* radiofrequency ablation, *RFA-EA* combination of RFA and EA, *AM* ablative margin, *LTP* local tumor progression

### Complications

No ablation-related mortality was observed. Major complications related to ablation were observed in four patients (4/73, 5.5%), including abdominal hemorrhage needing tube catheter in three and liver abscess in one. According to univariate analysis, no factors were associated with major complications after ablation for caudate lobe HCC (Table 6).

**Table 6:** Univariate and multivariate analysis of predictors of complication after caudate lobe ablation

Factors		Univariate	Multivariate		
		*P* value	HR	95%CI	*P* value
Gender(M/F)		1.000			
Age(≤60/>60y)		0.359			
Tumor type (naive/recurrent)		0.410			
Etiology (Hepatitis/others)		0.691			
Type (Naïve/recurrent)		0.299			
Surgery history of liver (Y/N)		1.000			
Liver cirrhosis (Y/N)		0.113			
ECOG performance status (0/1)		0.765			
Child-Pugh (A/B)		0.104			
BCLC stage of primary HCC (A/B)		0.453			
AFP (≥400/< 400 μg/L)		0.861			
PLT (≥100/< 100 × 10^9^/L)		1.000			
PT (≤14/> 14 s)		1.000			
ALB (≤35/> 35 g/L)		1.000			
ALT (≤40/> 40 IU/L)		0.164			
TB (≤17.1/> 17.1 mol/L)		1.000			
Tumor number (single/multiple)		1.000			
Location (Paracaval portion/ Caudate process/ Spiegel’s lobe)		0.727			
Tumor size (≤2 cm /> 2 cm)		0.691			
Puncture approach (LA/ RA/ combination approach)		0.724			
Treatment strategy	RFA/ EA/ RFA-EA	0.832			
	EA/RFA or RFA-EA	0.674			
	RFA or EA/RFA-EA	1.000			
AM (≥ 5 mm / < 5 mm)		0.443			

*HBV* hepatitis B virus, *HCV* hepatitis C virus, *ECOG* East Coast Oncology Group, *BCLC* Barcelona Clinic Liver Cancer, *AFP* alpha-fetoprotein, *PLT* platelets, *PT* prothrombin time, *ALB* albumin, *ALT* alanine aminotransferase, *TB* total bilirubin, *LA* left lobe approach, *RA* right intercostal approach, *EA* ethanol ablation, *RFA* radiofrequency ablation, *RFA-EA* combination of RFA and EA, *AM* ablative margin

## Discussion

Percutaneous ablation is a challenging procedure with evolving techniques for tumor in the caudate lobe, because the percutaneous puncture tract is narrow and the puncture route is surrounded by major vessels. In the present study, we reviewed our experience of ablation for HCC in the caudate lobe and evaluated the predictive factors of local treatment outcomes. Ultrasound-guided percutaneous ablation was feasible and effective for patients with caudate lobe HCC. Treatment strategy of EA was a significant prognostic factor of ICA in the patients with HCC in the caudate lobe. Meanwhile, tumor size > 2 cm increased the risk of LTP after ablation. Perhaps, our results would be helpful when making a treatment strategy for HCC in the caudate lobe of liver.

To date, a few studies focusing on percutaneous ablation for treatment of caudate lobe HCC have been published [11–13, 20, 21]. They all declared that ablation was an effective treatment modality for HCC in the caudate lobe [11–13, 20, 21]. Recently, Nishigaki et al. [13] compared treatment outcomes of RFA for HCC in the caudate lobe with those in the non-caudate lobe. It was described that HCC in the caudate lobe treated by RFA showed a high incidence of LTP. However, to our knowledge, no studies had analyzed the predictors of local treatment outcomes of caudate lobe tumors after ultrasound-guided percutaneous ablation.

We performed EA for tumors less than 2 cm without sufficient safety margin for thermal ablation. According to our results, treatment strategy of EA would increase the risk of ICA in caudate lobe tumors. Recently, Luo et al. [22] performed a systematic review and meta-analysis to compare the treatment effects of RFA, EA and RFA-EA for HCC. Their results showed that complete ablation rates were lower in patients performed with EA than those performed with RFA or RFA-EA, which was similar to our results for caudate lobe HCC.

In terms of the LTP after ablation for caudate lobe tumor, it was 38.5% at 3 years after the procedure. The rate was approximately three to four times as higher as that in patients with HCC located elsewhere in the liver, which was reported being approximately 10% at 3 years after ablation treatment [23–26]. Several factors such as tumor size [25], insufficient safety margin [27], pretreatment AFP level [28], and ALT level [26] had been reported as factors associated with LTP. However, in the present study focused on HCC in the caudate lobe, only tumor size > 2 cm was the risk factor associated with LTP. Indeed, a larger tumor had the tendency to develop LTP. Hereby, more meticulous ablation procedures may be needed for the treatment of caudate lobe tumor > 2 cm in size, with closer attention during the follow-up period.

On the other hand, although AM was not significantly an independent risk factor of LTP, 24.6% patients with AM < 5 mm were detected LTP whereas none patient with AM ≥5 mm had LTP. Although RFA-EA had been applied for some cases, only 9.7% of tumors achieved the aim of AM ≥5 mm. Indeed, it is quite difficult to increase the AM for caudate lobe HCC because of some factors, such as the tumor in caudate lobe located in the vicinity of major vessels is one of the factors. The coagulation ability of RFA is potentially impaired by the heat sink effect. The second factor is the difficulty in positioning the needle optimally in the caudate lobe. Not all sites in the caudate lobe can be targeted as inadvertent injury to important intra- and extrahepatic structures has to be avoided. This restricted puncture route is also a significant cause of an insufficient AM, possibly leading to LTP.

Although RFA-EA did not show significant improvement in the study, it remains unclear that whether such combination therapy is superior to monotherapy in the cases involving HCC in the caudate lobe. In theory, RFA-EA appears to be the optimal treatment strategy when considering RFA-EA or either RFA or EA alone. It has been reported that RFA-EA significantly improves OS and reduces the risk of LTP without increasing major complications [8]. However, there were no significant differences of ICA and LTP rates between RFA-EA and RFA or EA alone, possibly due to the small sample size. A future multicenter prospective study with a larger scale of patients and homogeneous background factors is warranted to evaluate the efficacy of ablation for malignant tumors located in the caudate lobe.

Given that RFA therapy as a curative treatment, but the results of DR and OS seemed to be poor. Perhaps, it was possibly due to the indication of ablation therapy in the institute. Moreover, in the present study, the most of tumors in the caudate lobe was recurrent after several sessions of prior treatments.

Complications may occur after ablation for tumors in the caudate lobe if insufficient care is taken during the procedure. It seems that an acceptable risk of complications could be achieved by careful positioning of the coagulation needle to avoid puncturing vital structures and to coagulate the needle tract when pulling out the needle to prevent bleeding and tumor dissemination. There was no mortality after RFA. Moreover, all the patients with major complications in the present study were fully recovered, without any serious adverse sequelae. Therefore, we believe that HCC located in the caudate lobe can be treated safely by percutaneous ablation if the procedure is performed cautiously.

There are several limitations in the present study. First, it was a retrospective study from a single institution, which might cause selection bias. Therefore, further prospective studies with a large scale from multi-center are required. Second, a small number of patients were involved. However, given that tumors in the caudate lobe are rare, data from small retrospective cohort studies can be served to build up the treatment evidence. Thirdly, the further comparison such as ablation versus liver resection for tumors or ablation for tumors in the caudate lobe versus those in the non-caudate lobe would improve the study.

## Conclusions

In conclusion, ultrasound-guided percutaneous ablation therapy is a feasible and effective treatment for a selected case with HCC in the caudate lobe. Tumor size of caudate lobe HCC larger than 2 cm increases the risk of LTP and intrahepatic tumor number is associated with DR after ablation. Further prospective randomized controlled trials are needed to validate these findings.

## Data Availability

The datasets used and/or analysed during the current study are available from the corresponding author on reasonable request.

## Abbreviation

AASLD: American Association for the Study of Liver Disease
AFP: Alpha-fetoprotein
ALB: Albumin
ALT: Alanine aminotransferase
AM: Ablative margin
CA: Complete ablation
CI: Confidence intervals
CT: Computed tomography
DR: Distant recurrence
EA: Ethanol ablation
EASL: European Association for the Study of Liver
ECOG: East Coast Oncology Group
HCC: Hepatocellular carcinomas
ICA: Incomplete ablation
LA: Left lobe approach
LTP: Local tumor progression
MRI: Magnetic Resonance Imaging
OS: Overall survival
PLT: Platelets
PT: Prothrombin time
RA: Right intercostal approach
RFA: Radiofrequency ablation
RFA-EA: Combination of RFA and EA
TACE: Transcatheter arterial chemoembolization
TB: Total bilirubin
